# Werner syndrome protein works as a dimer for unwinding and replication fork regression

**DOI:** 10.1093/nar/gkac1200

**Published:** 2022-12-30

**Authors:** Soochul Shin, Kwangbeom Hyun, Jinwoo Lee, Dongwon Joo, Tomasz Kulikowicz, Vilhelm A Bohr, Jaehoon Kim, Sungchul Hohng

**Affiliations:** Department of Physics and Astronomy, Institute of Applied Physics, Seoul National University, Seoul, Republic of Korea; Department of Biological Sciences, Korea Advanced Institute of Science and Technology, Daejeon, Republic of Korea; Department of Physics and Astronomy, Institute of Applied Physics, Seoul National University, Seoul, Republic of Korea; Department of Physics and Astronomy, Institute of Applied Physics, Seoul National University, Seoul, Republic of Korea; Section on DNA repair, National Institute on Aging, National Institutes of Health, Baltimore, MD 21224, USA; Section on DNA repair, National Institute on Aging, National Institutes of Health, Baltimore, MD 21224, USA; Department of Biological Sciences, Korea Advanced Institute of Science and Technology, Daejeon, Republic of Korea; Department of Physics and Astronomy, Institute of Applied Physics, Seoul National University, Seoul, Republic of Korea

## Abstract

The determination of the oligomeric state of functional enzymes is essential for the mechanistic understanding of their catalytic activities. RecQ helicases have diverse biochemical activities, but it is still unclear how their activities are related to their oligomeric states. We use single-molecule multi-color fluorescence imaging to determine the oligomeric states of Werner syndrome protein (WRN) during its unwinding and replication fork regression activities. We reveal that WRN binds to a forked DNA as a dimer, and unwinds it without any change of its oligomeric state. In contrast, WRN binds to a replication fork as a tetramer, and is dimerized during activation of replication fork regression. By selectively inhibiting the helicase activity of WRN on specific strands, we reveal how the active dimers of WRN distinctly use the energy of ATP hydrolysis for repetitive unwinding and replication fork regression.

## INTRODUCTION

The RecQ family of proteins including prototypic *Escherichia coli* RecQ, and five *Homo sapiens* helicases (RECQL1, BLM, WRN, RECQL4 and RECQL5) is highly conserved from bacteria to human (1). They are known to play crucial roles in genome maintenance, and mutations in RECQ4, BLM, and WRN give rise to genetic disorders associated with inherent genomic instability and cancer predisposition (2–6). For instance, Werner syndrome caused by mutations of *WRN* is characterized by features of premature aging and a significantly increased incidence of cancer (7). Cells derived from Werner syndrome patients are characterized by high frequency of chromosomal insertions, deletions and translocations (8,9).

RecQ proteins are ATP-dependent motor proteins that have diverse biochemical activities. They have a helicase activity to unwind not only double-stranded DNA but also non-canonical structures such as forks, bubbles, G-quadruplexes and Holliday junctions (10–12). RecQ proteins such as BLM and WRN have the activity of replication fork regression, one of the major pathways for reactivating stalled DNA replication. In addition to these ATP-dependent activities, WRN exhibits 3′-5′ DNA exonuclease (13) and strand annealing activities (14,15). These diverse activities of RecQ proteins appear to be cooperatively used to efficiently deal with pathological intermediate structures associated with DNA replication, repair and recombination (11,16–18). The helicase and exonuclease activities of WRN likely function coordinately in DNA unwinding (19) and fork regression (20). The helicase and annealing activities of WRN seem to catalyze strand exchange (14), which may be operational in homologous recombination.

It has been suggested that the diverse biochemical activities of RecQ helicases are related to their quaternary structures (21), and there have been efforts to determine the stoichiometry of various RecQ helicases related to their biochemical activities. Using chromatography and EM (Electron Microscopy) methods, for example, Karow *et al.*, reported that BLM formed hexamers in the absence of DNA substrates (22). Using DLS (Dynamic Light Scattering), Xu *et al.* reported that multimeric BLM likely is in a hexameric form, which is then dissociated upon ATP hydrolysis probably into a dimer or monomer (23). They further suggested that the active form of BLM is a monomer on various substrates such as DNA duplexes, Holliday junctions and G-quadraplexes. Using AFM (atomic force microscope), EM and DLS, Gyimesi *et al.* reported that BLM existed mainly as a monomer in solution in the absence of ATP and DNA substrates (24). They further showed that monomeric BLM binds to and is active on various DNA substrates except complex DNA structures such as Holliday junctions on which BLM is dimerized. Vindigni's group used chromatography and DLS, and reported that human RecQ1 exists as a dimer in solutions (25). Three years later, the same group used TEM together with chromatography, and reported that strand annealing in the absence of ATP is performed by hexameric or pentameric RecQ1 whereas the unwinding is performed by monomeric or dimeric RecQ1, formed by dissociation of higher oligomeric forms of RecQ1 upon ATP binding (26). In 2011, the same group used DLS together with chromatography, and reported that RecQ1 existed as a mixture of dimeric and tetrameric forms in solution (27). They further showed that dimer formation promoted DNA unwinding whereas tetrameric RecQ1 was necessary for Holliday junction resolution and strand annealing activity. Using chromatography, Huang *et al.* reported that WRN exists as a trimer in solution (28). Using EM, Compton *et al.* reported that WRN existed mainly as a dimer in solution, but that a tetrameric form became dominant on substrates such as replication fork and Holliday junctions (29). Liu *et al.* reported that monomeric RECQL is more abundant, and more active than dimeric and tetrameric forms in living cells (30). In these works, however, the coupling of stoichiometry and protein activities were not directly studied, but done separately, making the correlation between these parameters ambiguous, especially when oligomeric states are heterogeneous and intermittent.

Here, we determine the oligomeric states of WRN that are active for DNA unwinding and replication fork regression using single molecule FRET (31) combined with single molecule subunit counting (32,33); the number of protomers of WRN oligomers is determined by counting the photobleaching steps of the GFP signal tagged to WRN, and the biochemical activities of WRN are confirmed by measuring FRET signals. Our data reveal that a dimeric WRN preferentially binds to and unwinds a forked DNA. On the other hand, a tetrameric WRN binds to a model replication fork and is then converted into a dimer during the activation of replication fork regression. We further show that each protomer of the dimeric WRN is used distinctly for repetitive unwinding and repetitive replication fork regression.

## MATERIALS AND METHODS

### Protein preparation

Human RPA was expressed from plasmid vector p11d-tRPA (gift from Dr Marc S. Wold) (34) in *Escherichia coli* BL21 (Novagen) and purified as previously described (35). Human WRN was expressed and purified using a baculovirus/Sf9 insect cell system as previously described (36). Briefly, cDNA encoding full-length WRN was generated by RT-PCR from total RNA extracted from HEK293T cells and subcloned into pFASTBAC1 plasmid (Thermo Fisher Scientific) with a GFP and an N-terminal FLAG-tag (Supplementary Figure S1). Baculoviruses were generated according to the manufacturer's instructions (Thermo Fisher Scientific) and infected into Sf9 insect cells. After 3 days, cell extracts were prepared in lysis buffer (20 mM Tris–HCl pH 7.9, 300 mM KCl, 2.5 mM MgCl_2_, 0.25 mM EDTA, 15% glycerol, 0.1% NP-40, 1.2 mM DTT, and 0.5 mM PMSF) and clarified extracts were subjected to affinity purification on M2 agarose beads (Sigma-Aldrich). After extensive washing with wash buffer (20 mM Tris–HCl pH 7.9, 150 mM KCl, 2 mM MgCl_2_, 0.2 mM EDTA, 15% glycerol, 0.1% NP-40, 1 mM DTT and 1 mM PMSF), protein was eluted with wash buffer containing 0.25 mg/ml FLAG peptide. We prepared two versions of GFP-tagged WRN (f:GFP-WRN and f:WRN-GFP as presented in Supplementary Figure S1A), but f:GFP-WRN was solely used for the experiments because f:WRN-GFP did not exhibit the unwinding and fork regression activities in the single-molecule experiments. SDS-PAGE images of f:GFP-WRN and f:WRN-GFP are shown in Supplementary Figure S1B.

### Preparation of DNA/RNA substrates

DNA strands and RNA strands were purchased from Integrated DNA Technologies (IDT, Coralville, IA) and ST Pharm (Seoul, Korea), respectively. For dye labeling, 0.5 mM DNA(or RNA) modified with an amino C6 dT(or rU in RNA case) was incubated with 10 mM amine-reactive fluorophore in a reaction buffer (100 mM Na_2_BO_7_ pH 8.5) over 12 h. The excess dye was removed using ethanol precipitation. Forked DNAs were prepared by mixing top (80 nt, 200 μM, 2 μl) and bottom (74 nt, 200 μM, 1 μl) strands, and slowly cooling down from 95°C to 4°C with a cooling rate of –1°C per minute. Model replication forks were sequentially annealed as follows. First, the leading and lagging arms were annealed separately by cooling the mixture of the parent and daughter strands from 90°C to 4°C with –1°C/min velocity. Next, the leading and lagging arms were mixed and cooled down from 50°C to 4°C with –1°C/min velocity. The annealing reaction was performed in a 10 mM Tris–HCl pH 8.0 buffer containing 50 mM NaCl.

### Single-molecule FRET experiment

Quartz slides and glass coverslips were cleaned with piranha solution (mixture of sulfuric acid and hydrogen peroxide) and then were coated with 40:1 mixture of poly-ethylene glycol (PEG) and biotin-PEG. A simple microfluidic sample chamber (volume: ∼20 μl) was made by assembling PEG-coated quartz slide and glass coverslip using double-sided tape. Plastic tubing and syringe pump (Fusion 100, Chemyx Inc.) were used for automatic buffer exchange during measurement. Flow rate of 200 μl/s was used so that buffer exchange time was less than time resolution of the experiment (0.4 s). The DNA samples were immobilized on the PEG-passivated surface via biotin–streptavidin interaction and then were incubated with 26 nM wild type WRN or 24 nM GFP-WRN in 10 mM Tris–HCl pH 8.0 buffer containing 1 mM MgCl_2_, 100 ng/ml BSA, 1 mM DTT and 1 mM trolox, or containing 50 mM NaCl, respectively, for 5 min. While an imaging buffer (10 mM Tris–HCl pH 8.0, 1 mM MgCl_2_ with an oxygen scavenger system: 0.4 % (w/v) glucose, 1 mM Trolox, 1 mg/ml glucose oxidase, 0.04 mg/ml catalase) with or without 1 mM ATP was used in Cy3/Cy5 signal detection to reduce their photobleaching, a standard buffer (10 mM Tris–HCl pH 8.0, 50 mM NaCl) with or without 1 mM ATP-Mg^2+^ was used in GFP photobleaching step counting measurements to avoid inactivation of GFP. All experiments were carried out at room temperature. Fluorescence signals were obtained using a prism-type total internal reflection fluorescence microscope equipped with electron multiplying charge coupled device (EM-CCD; Ixon + DU897BV, Andor Technology). Three lasers, 473 nm (Cobolt Blues 50, Cobolt), 532 nm (Compass 215M, Coherent), 640 nm (Cube 640C, Coherent), were used as excitation sources. A homemade program written in Visual C++ (Microsoft) was used for data acquisition.

### Data analysis

For photobleaching step counting, firstly we localized molecules using both Cy3 and Cy5 signals, and then GFP fluorescence intensity time traces were obtained from these molecules. During data analysis, 11 × 11 sub-image near region of interest (ROI) was checked to confirm that there was only one molecule in ROI. After obtaining photobleaching step number histograms the histograms were normalized and fitted to binomial distributions *X ∼ B* (*n, p*). For binomial fitting, *n* was fixed to maximum number of observed bleaching steps. All data analysis was performed by using homemade programs written in IDL (ITT, peak finding, trace making), MATLAB (MathWorks, visualization data, counting, dwell time), and Origin (OriginLab, fitting).

## RESULTS

### Dimeric WRN preferentially binds to a forked DNA while tetrameric WRN binds to a model replication fork

**Figure 1.**
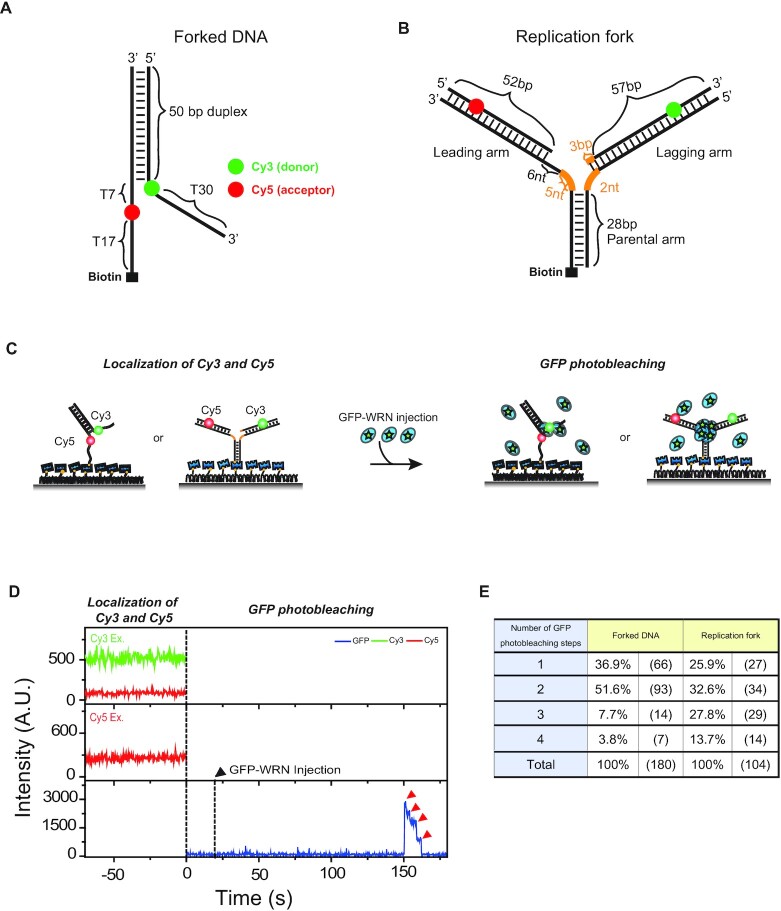
Oligomeric states of WRN that differentially bind to a forked DNA and a replication fork. (**A**) A design of a forked DNA. (**B**) A design of a model replication fork. Orange lines represent heterologous bases. (**C**) The experimental procedure to assess the oligomeric states of WRN. (**D**) Representative fluorescence intensity time traces of Cy3 (green), Cy5 (red) and GFP (blue). After Cy3 and Cy5 imaging for co-localization, GFP-tagged WRN was injected at *t* = 0. The first dashed line indicates the timing of buffer exchange from the Cy3/Cy5 imaging buffer with oxygen scavenging system to the GFP imaging buffer without oxygen scavenging system. The second dashed line (black arrow) indicates the injection of GFP-tagged WRN. Red arrow indicates the photo-bleaching steps of GFP. (**E**) Distribution of photobleaching steps for forked DNA, and model replication fork.

To determine the oligomeric states of WRN by counting photobleaching steps of GFP, we prepared GFP-tagged WRN (Materials and Methods). This is an approach that has been widely used to determine the oligomeric states of other proteins (Supplementary Note S1). The WRN with GFP tagged at the N-terminal has also been used in a number of previous studies (37–42). We further confirmed that GFP-tagged WRN has the same unwinding and fork regression activities as the wild type WRN by using the single-molecule assays well established in our previous studies (43–45) even though the populations of active molecules were apparently reduced with a GFP tag (Supplementary Figure S2). As binding substrates of GFP-tagged WRN, we prepared two DNA substrates: a forked DNA (Figure 1A), and a model replication fork (Figure 1B). The forked DNA has a Cy3 (donor) and a Cy5 (acceptor) in their single-stranded overhangs, and biotin at the 5′-end of the single-stranded overhang. In this scheme, a FRET decrease reports the unwinding events by WRN. The model replication fork is composed of a 28-bp parental arm, a leading arm with a 52-bp duplex region and an 11-nt single-stranded gap at the junction, and a lagging arm with a 60-bp duplex and a 2-nt single-stranded gap at the junction. To prevent spontaneous branch migration, we introduced heterologous nucleotides near the junction (orange, Figure 1B). The model replication fork has a Cy3 (donor) and a Cy5 (acceptor) as a FRET pair, and biotin at the 5′- of the parental arm. In this experimental scheme, a FRET appearance indicates the formation of a Holliday junction, and simultaneous disappearance of Cy3 and Cy5 signals represents the dissociation of the daughter duplex after the completion of replication fork regression. Similar DNA substrates were previously used to study repetitive unwinding (43), and replication fork regression of WRN (45).

To assess the oligomeric states of WRN in solution, and their possible preference for specific DNA binding substrates, we performed single-molecule fluorescence experiments using total internal reflection fluorescence microscopy to observe the real-time binding events of GFP-tagged WRN to DNA substrates. First, we immobilized DNA substrates on a quartz surface via biotin-streptavidin interaction and localized the substrates by imaging Cy3 and Cy5. Subsequently, we added GFP-tagged WRN into the detection chamber and monitored the GFP signal at the localized DNA substrates (Figure 1C). Since dissolved oxygen molecules rapidly photobleach cyanine dyes, Cy3 and Cy5 imaging was done using the oxygen scavenger system (31). GFP imaging was done without the oxygen scavenger system because GFP becomes inactive in the absence of oxygen (46–48).

Representative intensity time traces of Cy3, Cy5 and GFP on DNA substrates are shown in Figure 1D. The presence of both Cy3 and Cy5 signals indicated that the DNA substrates were intact. After the injection of GFP-tagged WRN, its binding events could be monitored as GFP signal jumps. The binding occurred just once, and no further binding was observed from the same molecule over a period of 30 min (Supplementary Figure S3). This observation indicates that the WRN-DNA interaction is quite stable, and no further oligomerization process occurs via successive binding of WRN to DNA substrates.

After binding, GFP photobleached in a stepwise manner (bottom, Figure 1D). For both the forked DNA and the model replication fork, diverse photobleaching steps were observed with a maximum number of 4 (Figure 1E). The distributions of the photobleaching steps, however, were quite different; the counts for n = 3 and 4 were significantly increased in the model replication fork compared to the forked DNA. To test the homogeneity of the oligomeric states of WRN, the distributions were fitted to binomial distributions. Since the photobleaching of some subunits are not observed mainly due to the existence of nonfluorescent GFPs (32,49), the photobleaching step distribution is expected to be binomial for a homogeneous population (Supplementary Note S1). The photobleaching step distribution of the forked DNA was not well fitted to a binominal distribution *X ∼ B* (*n, p*) with *n* = 4 (Supplementary Figure S4A), suggesting that the oligomeric states of WRN binding to the fork DNA were heterogeneous. The conclusion was not affected by the presence of 1 nM RPA in solution (Supplementary Figure S5). On the other hand, the photobleaching step distribution of the model replication fork was well fitted to a binominal distribution *X ∼ B* (*n, p*) with *n* = 4 (Supplementary Figure S4B). We also confirmed that the photobleaching step distributions were not significantly affected by salt or WRN concentration (Supplementary Figure S6). These observations are consistent with previous reports that the oligomeric states of WRN are diverse in solution, and different oligomeric states of WRN have varying preference for distinct substrates (29,50). Based on our data, it seems reasonable to conclude that dimeric WRN prefers a forked DNA rather than a model replication fork as a binding substrate, whereas tetrameric form of WRN prefers model replication fork to a forked DNA as a binding substrate.

### Dimeric WRN unwinds forked DNA

**Figure 2.**
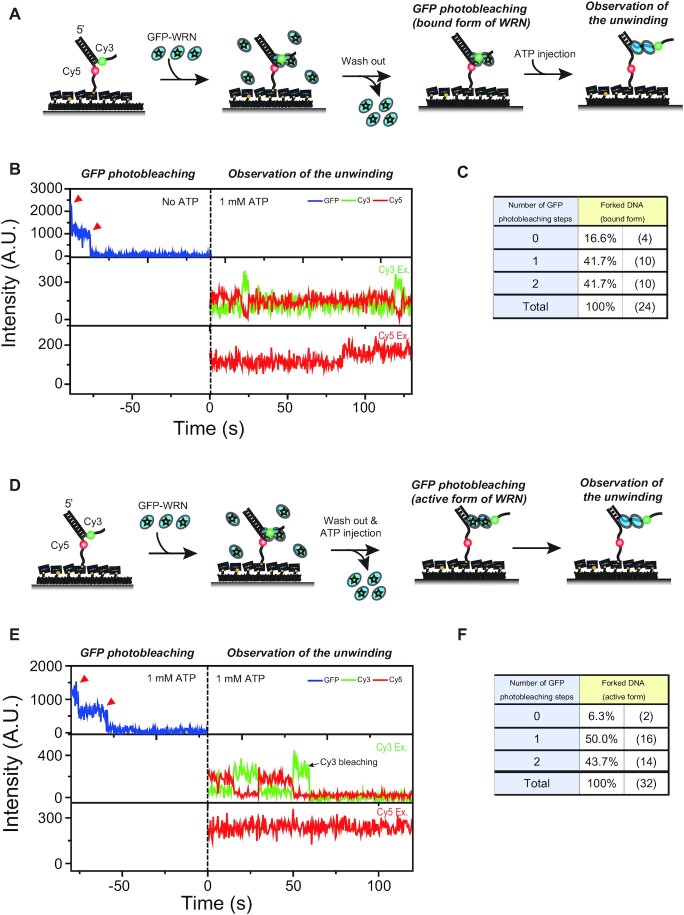
Unwinding of forked DNA by WRN dimer (**A**) The experimental scheme to check the unwinding activity of WRN after observing the oligomeric state of WRN binding to the forked DNA. (**B**) Representative fluorescence intensity time traces of the experiment (A). Photobleaching steps of GFP were counted in the absence of ATP. After the ATP injection, repetitive unwinding of the forked DNA by WRN was observed. (**C**) Distribution of photobleaching steps of experiment (B). (**D**) The experimental scheme to observe the oligomeric state of WRN that are repetitively unwinding the forked DNA. (**E**) Representative fluorescence intensity time traces of the experiment. Photobleaching steps of GFP were counted in the presence of ATP, and the unwinding activity of WRN was checked using FRET in the Cy3/Cy5 imaging buffer. (**F**) Distribution of photobleaching steps of experiment (E).

After characterizing the oligomeric states of WRN that bind to a forked DNA and a model replication fork, we studied their catalytic activities. To characterize the unwinding activity of oligomeric states of WRN on the forked DNA, we first used the experimental procedure described in Figure 2A. DNA–WRN complexes were made by incubating immobilized forked DNA with 24 nM GFP-tagged WRN and 1 nM RPA. It was reported that RPA enhances the unwinding activity of WRN (43). After washing out unbound proteins, the number of GFP photobleaching steps was counted. Finally, the unwinding activity of WRN was checked using Cy3–Cy5 FRET after the injection of 1 mM ATP-Mg^2+^. Figure 2B shows the representative fluorescence intensity time traces of GFP, Cy3, and Cy5. As previously reported with wild type WRN (43), we detected the repetitive unwinding of forked DNA using GFP-tagged WRN; FRET efficiency drops when the forked DNA is partially unwound whereas it recovers to its original value when the forked DNA is rewound (Supplementary Figure S7A). Although we observed the repetitive unwinding events using GFP-tagged WRN, we noticed that its efficiency was hampered compared to that of wild type WRN (Supplementary Figure S2), probably due to the steric hindrance of GFP on the forked DNA substrate. When we counted the photobleaching steps of the molecule with the unwinding activity only, the maximum number was two (Figure. 2C). Furthermore, the photobleaching step distribution was well fitted to a binominal distribution *X ∼ B* (*n, p*) with *n* = 2, *p* = 0.63 and χ^2^ = 0.013 (Supplementary Figure S4C), suggesting a homogeneous population. These results are contrasted with the results of Figure 1E, in which the maximum number was four and the distribution was not well fitted to a binomial distribution. Based on these considerations, we conclude that only the dimeric WRN can unwind the forked DNA. While the tetrameric form rarely binds to forked DNA as shown in Figure 1, it does not have the unwinding activity of the forked DNA.

For BLM and RecQ1, it has been reported that ATP binding or hydrolysis induces the change of their oligomeric states (23,26). To test this for WRN, we set up a different experimental scheme described in Figure 2D. After making DNA-WRN complexes by incubating DNA with GFP-tagged WRN and RPA, unbound proteins were washed out. Next, DNA-WRN complexes were incubated with 1 mM ATP-Mg^2+^ for 2 min to activate the repetitive unwinding of the forked DNA, and then the number of GFP photobleaching steps was counted. Finally, the unwinding activity of WRN was checked using Cy3-Cy5 FRET. Representative fluorescence intensity time traces are shown in Figure 2E, and the distribution of GFP photobleaching steps is shown in Figure 2F. The distribution was similar to that in Figure 2C, and the distribution was well fitted to the binominal distribution *X ∼ B* (*n, p*) with *n* = 2, *p* = 0.67 and χ^2^ = 0.027 (Supplementary Figure S4D). Therefore, the dimeric form of WRN can unwind the forked DNA without being converted into the monomeric form by ATP.

### Tetrameric WRN on a replication fork is converted into a dimer for replication fork regression

**Figure 3.**
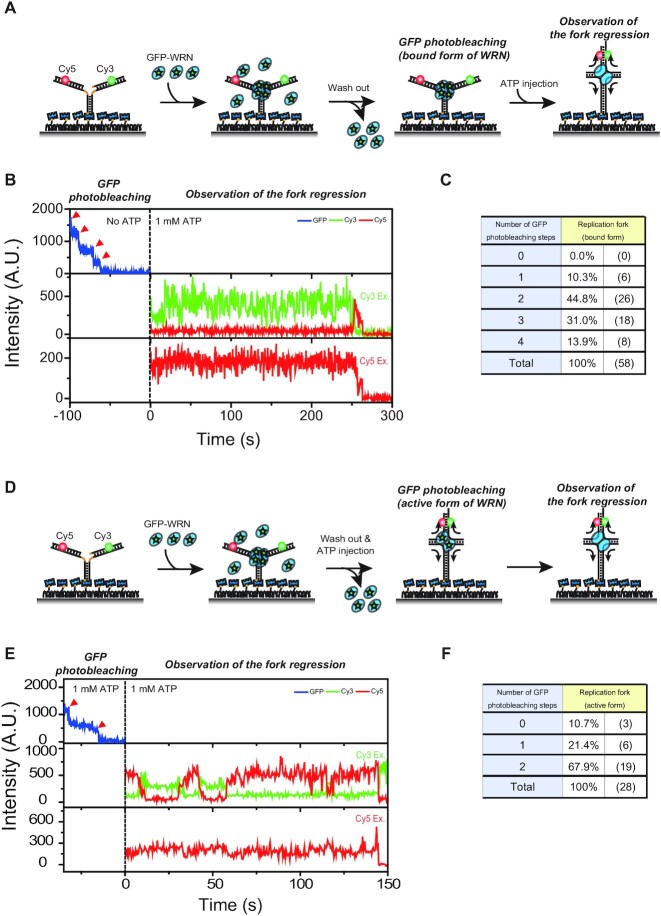
Tetramer-to-dimer transition of WRN for replication fork regression. (**A**) The experimental scheme to check the unwinding activity of WRN after observing the oligomeric state of WRN binding to the model replication fork. (**B**) Representative fluorescence intensity time traces of the experiment (A). Photobleaching steps of GFP were counted in the absence of ATP. After the ATP injection, replication fork regression was observed. (**C**) Distribution of photobleaching steps of experiment (B). (**D**) The experimental scheme to observe the oligomeric state of WRN that is doing repetitive replication fork regression. (**E**) Representative fluorescence intensity time traces of the experiment (D). Photobleaching steps of GFP were counted in the presence of ATP, and the replication fork regression activity of WRN was checked using FRET in the Cy3/Cy5 imaging buffer. (**F**) Distribution of photobleaching steps of experiment (E).

To test whether the tetrameric form of WRN can perform replication fork regression, we first performed an experiment described in Figure 3A. DNA-WRN complexes were made by incubating immobilized replication forks with GFP-tagged WRN. After washing out unbound WRN, the number of GFP photobleaching steps was counted. Finally, the replication fork regression activity of WRN was checked using Cy3–Cy5 FRET after injection of 1 mM ATP-Mg^2+^. Figure 3B shows representative fluorescence intensity time traces of GFP, Cy3 and Cy5. As previously reported for wild type WRN (45), we observed replication fork regression activity using GFP-tagged WRN; FRET efficiency jumps when the replication fork is regressed into a four-way junction, and fluorescence signals disappear when the daughter strands dissociate from the substrate (Supplementary Figure S7B). The maximum number of GFP photobleaching steps of the molecules exhibiting the repetitive replication fork regression activity was four (Figure 3C), and the photobleaching step distribution was well fitted to a binominal distribution *X ∼ B* (*n, p*) with *n* = 4, *p* = 0.61 and χ^2^ = 0.075 (Supplementary Figure S4E). Therefore, tetrameric WRN binding to a replication fork can perform replication fork regression. However, this does not require that the active form of WRN is tetrameric because the oligomeric state of WRN can change in the presence of ATP whereas the photobleaching steps of GFP in Figure 3C were counted in the absence of ATP; it has been reported that the oligomeric state of RecQ helicases changes in the presence of ATP (51,52).

To test whether the oligomeric state of WRN changes after addition of ATP, we used a model replication fork with heterologous regions at the distal ends of the leading and lagging arms (Supplementary Table S1) so that replication fork regression occurs repetitively (45), and performed the experiment described in Figure 3D. After making DNA–WRN complexes by incubating DNA with GFP-tagged WRN, unbound WRN was washed out. Next, DNA–WRN complexes were incubated with 1 mM ATP-Mg^2+^ for 2 minutes to activate WRN, and then the number of GFP photobleaching steps was counted. Finally, the activity of WRN was checked using Cy3–Cy5 FRET. Representative fluorescence intensity time traces are shown in Figure 3E, and the distribution of GFP photobleaching steps of active WRN is shown in Figure 3F. Surprisingly, the maximum photobleaching steps observed in this case was two, and the distribution was well fitted to a binominal distribution *X ∼ B* (*n, p*) with *n* = 2 and *p* = 0.81, and χ^2^ = 0.176 (Supplementary Figure S4F). Therefore, the tetrameric WRN binding to a model replication fork was converted to a dimer upon ATP addition. To test whether ATP hydrolysis is required for the tetramer-to-dimer transition or not, we incubated DNA–WRN complex with ATPγS instead of ATP, counted the GFP photobleaching steps, and checked the WRN activity after injecting ATP (Supplementary Figure S8). In this case the maximum number of GFP photobleaching steps observed from the active WRN was four (Supplementary Figure S8), indicating that the tetramer-to-dimer transition of WRN is an active process requiring ATP hydrolysis.

### The rewinding of the forked DNA does not require the motor activity of WRN

**Figure 4.**
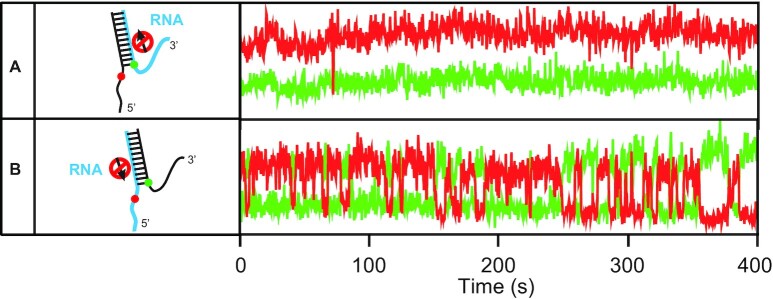
Unwinding activity of WRN on DNA/RNA hybrid substrates. (**A**) DNA/RNA hybrid sample with a 3′-overhang RNA strand (left). The RNA region is indicated by cyan. Representative fluorescence intensity time traces of Cy3 (green) and Cy5 (red) in the presence of 1 mM ATP and 1nM RPA (right). No molecule (0/698) shows unwinding behavior. 26 nM wild type WRN was used. (**B**) DNA/RNA hybrid sample with a 5′-overhang RNA strand (left). Representative intensity time traces of Cy3 (green) and Cy5 (red) in the presence of 1 mM ATP and 1nM RPA (right). 30.9% (224/725) of molecules show unwinding behavior. 26 nM wild type WRN was used.

We showed that dimeric WRN is an active form for both unwinding and replication fork regression, but it still remains unclear how ATP hydrolysis energy is used in repetitive unwinding and fork regression by the two protomers of the dimeric WRN. To address this question, we utilized the fact that WRN is a motor protein with a 3′–5′ directionality which has a strong ATP helicase activity on DNA substrates, but not on RNA substrates (53). This permits the selective inhibition of the motor activity of WRN on specific strands by replacing a DNA strand with an RNA strand. First, we prepared two kinds of DNA/RNA hybrid forks: one with a 3′-overhang RNA strand (left, Figure 4A), and another with a 5′-overhang RNA strand (left, Figure 4B). Repetitive unwinding was persistently observed in the case of 5′ RNA overhang substrate (right, Figure 4B) whereas no unwinding was observed in the case of 3′ RNA overhang (right, Figure 4A). These observations indicate that only the unwinding process of the forked DNA requires the motor activity of WRN while the rewinding process does not; if the motor activity of WRN is also required for the rewinding process, the unwound forked DNA cannot be rewound in the case of the forked substrate with a 5′-overhang RNA strand. This conclusion is contradictory to the previous report on BLM (54), but consistent with the work on HIM-6 (55). However, we cannot exclude the possibility that the rewinding mechanisms of WRN on DNA duplexes and on DNA–RNA hybrids differ.

### Both protomers of the dimeric WRN are used for replication fork regression

**Figure 5.**
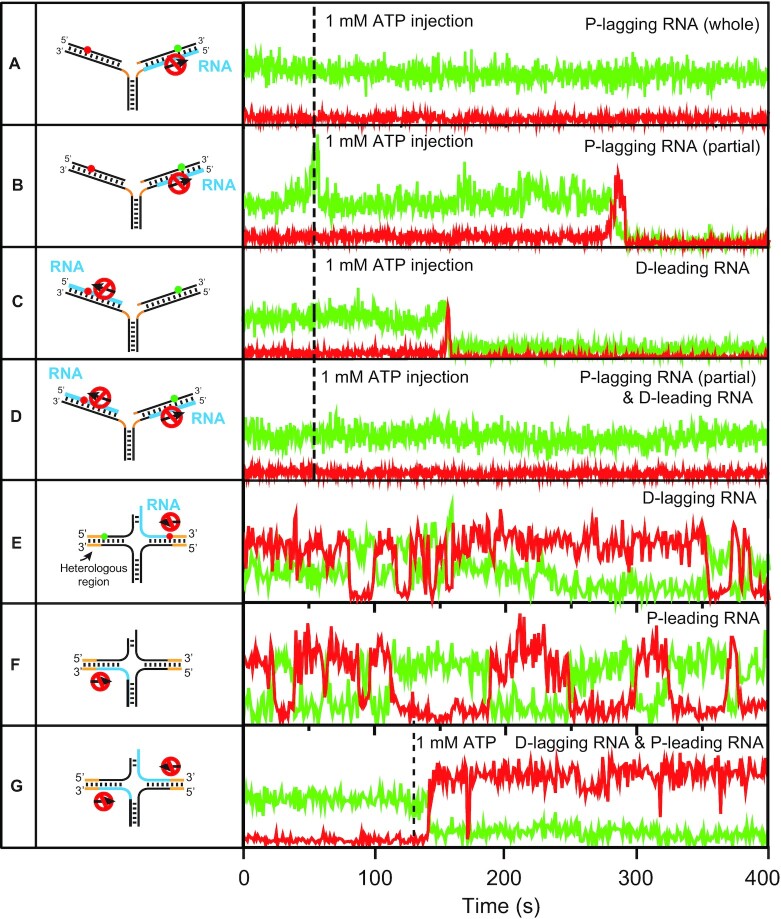
Replication fork regression activity of WRN on DNA/RNA hybrid substrates. (A–G, left) Schematics of sample designs. The RNA, and heterologous regions are indicated by cyan, and orange, respectively. (A–G, right) Representative fluorescence intensity time traces of Cy3 (green) and Cy5 (red) (right). Dashed lines indicate the 1 mM ATP injection timing for the initiation of replication fork regression. In (E) and (F), experiments were performed in the presence of 1 mM ATP. 26 nM wild type WRN was used in all experiments. The number of molecules showing activities are (**A**) (0/780), (**B**) (190/625), (**C**) (250/732), (**D**) (0/712), (**E**) (175/633), (**F**) (158/615) and (**G**) (208/705).

To address the question of how ATP hydrolysis energy is used by both protomers of the dimeric WRN for replication fork regression, we first inhibited the motor activity on the lagging arm by replacing the whole duplex region of the parental lagging strand with RNA (left, Figure 5A). In this case, replication fork regression was not observed (right, Figure 5A). However, when the initial part of the parental lagging strand that base-pairs with the leading parental strand was DNA, and the remaining part was RNA (left, Figure 5B), successful replication fork regression was observed (right, Figure 5B). These observations indicate that at least one protomer of the dimeric WRN operates on the lagging arm, and its motor activity is critical for the formation of a Holliday junction, but not for the directional migration of the Holliday junction. To test the role of the motor activity of WRN on the leading arm, we replaced the entire daughter strand of the leading arm with RNA (left, Figure 5C). Successful replication fork regression events were observed (right, Figure 5C). However, no replication fork regression was observed (right, Figure 5D) when the translocation activity of WRN on both the parental lagging strand and the daughter leading strand were inhibited (left, Figure 5D). The observations in Figure 5A–D can be explained by assuming that either one of the two protomers of the dimeric WRN is sufficient for the migration of the Holliday junction.

To further confirm this assumption, we performed similar experiments with model replication forks with heterologous regions at the distal ends of the leading and lagging arms that exhibit repetitive replication fork regression. When either the daughter lagging strand or the parental leading strand was replaced with RNA (left, Figure 5E, F), repetitive replication fork regression was observed (right, Figure 5E, F). When both of them were replaced with RNA (left, Figure 5G), however, the replication fork regression occurred once, and the replication fork was not recovered (right, Figure 5G).

## DISCUSSION

Biomolecules such as membrane proteins, topoisomerases, and helicases tend to form oligomers to be functional, and determination of the oligomeric state of biomolecules is a crucial step towards mechanistic insight into their biological activities. Various biophysical techniques at the ensemble level (size exclusion column, EM, AFM, DLS and crystallography) and at the single-molecule level (single-molecule photobleaching counting) have been used to determine the oligomeric states of the active forms of biomolecules. In these approaches, however, studies of protein stoichiometry and their activities are not directly coupled, but done separately, making the correlation between the protein stoichiometry and their activity ambiguous especially when oligomeric states are heterogeneous and dynamic.

We used single-molecule multi-color imaging to study the correlation of the oligomeric state with their unwinding, and replication fork regression activities of the same WRN molecule, and revealed the following. The oligomeric state of WRN in solution is heterogeneous with dimeric and tetrameric forms, and the dimeric WRN and the tetrameric WRN preferentially recognize a forked DNA and a stalled replication fork, respectively.

We discovered that the tetrameric WRN binding to a model replication fork is converted into a dimer using the ATP hydrolysis energy, whereas the dimeric WRN binding to a forked DNA is not converted into a monomer upon ATP addition. It was previously reported for RECQL1 (26) and BLM (23) that ATP causes an equilibrium shift toward the smaller form in solution. Our finding is distinct from these observations as the change of oligomeric states of WRN is DNA substrate-specific.

Various RecQ helicases have been shown to exhibit repetitive unwinding behavior (43,54,56–58), but the mechanisms were not clearly understood. Two possible models for the rewinding step are: (i) active translocation along the displaced strand after strand-switching and (ii) passive sliding back along either the translocated strand or the displaced strand. Based on our finding that the rewinding of the forked DNA does not require the motor activity of WRN, we can exclude the active translocation model (i) for the rewinding phase of the repetitive unwinding of WRN. However, this does not address the sliding back model.

During replication fork regression, the unwinding of the leading and lagging arms and the base pairing between the daughter strands and between parental strands occur simultaneously, but it is not known which strand is used as a translocation track of WRN. WRN is a motor protein with a 3′-5′ directionality, and either the parental lagging strand or the daughter leading strand can be a track for replication fork regression. We revealed that the motor activity of WRN on either of the two strands is sufficient for replication fork regression. It is conceivable that the two protomers of the dimeric WRN simultaneously translocate in opposite directions on either the parental lagging strand or the daughter leading strand (the parental lagging strand or the daughter leading strand are actually pulled into the junction because the WNR dimer remains intact) while base-paired parental and daughter arms are spinning out from the junction. Branch migration of the Holliday junction is not energy-demanding and can occur spontaneously. Therefore, it seems reasonable that WRN can be fully functional even when one of the two protomers of the dimeric WRN is not functional, as it is expected to happen in a heterozygous carrier individual with WRN syndrome. This observation also provides a molecular basis for explaining why WRN syndrome, like most autosomal recessive diseases, requires two mutant copies of the *WRN* gene to express the phenotype, and that the carriers with only one mutant allele appear normal.

**Figure 6.**
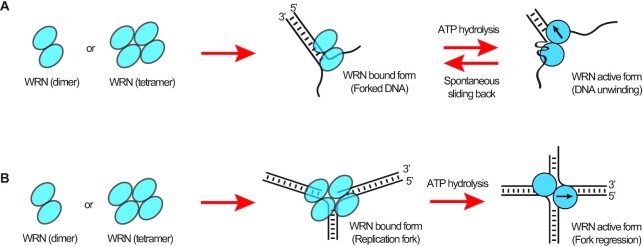
Mechanistic models of repetitive unwinding, and replication fork regression. (**A**) In solution, both dimeric and tetrameric forms of WRN coexist. Dimeric WRN preferentially binds to the forked DNA, and unwinds it with one protomer translocating on the 3′-overhang strand and the other anchoring on the 5′-overhang strand. As the unwinding progresses, mechanical stress accumulates, and rewinding abruptly occurs via the sliding back mechanism once a threshold is passed. (**B**) Tetrameric WRN preferentially binds to a stalled replication fork, and is converted into a dimer for the activation of the replication fork regression. During branch migration, the two protomers of the dimeric WRN translocate on either the parental lagging strand or the daughter leading strand.

In summary, we suggest mechanistic models for the repetitive unwinding and replication fork regression of WRN. Firstly, WRN binds to a forked DNA as a dimer, and unwinds it with one protomer translocating on the 3′-overhang strand and the other anchoring on the 5′-overhang strand (Figure 6A). As the unwinding progresses, mechanical stress accumulates, and rewinding spontaneously happens when the translocation protomer releases the tracking strand once a threshold of mechanical stress is passed. On the other hand, WRN binds to a stalled replication fork as a tetramer, and is converted into a dimer for the activation of the replication fork regression (Figure 6B). During branch migration, the two protomers of the dimeric WRN translocate on either the parental lagging strand or on the daughter leading strand. In this way, the dimeric WRN can be fully functional even when one of the two protomers is not active.

The discovery of fluorescent proteins has revolutionized life sciences. We used GFP-tagged WRN for this study, and observed that GFP tagging did not abolish the unwinding and fork regression activities of WRN, but their efficiencies were apparently reduced. The results were interpreted as that the steric hindrance of GFP reduced the efficiencies of the unwinding and fork regression activities of WRN but did not affect their mechanisms. However, there always exists a possibility that the relatively large size of GFP can cause artifacts. Photophysical properties of fluorescent proteins are not ideal for single-molecule studies compared to organic dyes, hampering accurate counting of photobleaching steps. We expect further studies using organic dyes with a smaller size and improved photophysical properties will resolve the issue.

This study cannot exclude the possible existence of other oligomeric forms of WRN in solution such as trimer or hexamer as we observed binding/active forms (not free form) of WRN only on forked DNA and model replication forks. The oligomeric states of WRN for the exonuclease and strand annealing activities are not addressed in this work, either. Further studies using more extensive DNA substrates such as DNA bubbles, G-quadruplexes will be able to clarify the issue.

## DATA AVAILABILITY

All data are available from the corresponding authors upon reasonable request.

## Supplementary Material

gkac1200_Supplemental_File
