# Supplementary material for: Werner syndrome protein works as a dimer for unwinding and replication fork regression
Source: Nucleic Acids Res. 2022 Dec 30;51(1):337–48. doi: 10.1093/nar/gkac1200 (PMC9841404; doi:10.1093/nar/gkac1200)
Supplement: gkac1200_Supplemental_File [file gkac1200_supplemental_file.pdf]

Supplementary Data

Werner syndrome protein works as a dimer for unwinding and replication fork regression

Contents

Supplementary Figure S1. Purification of WRN.

Supplementary Figure S2. Comparison of the unwinding and fork regression activities of wild-type WRN and GFP-WRN.

Supplementary Figure S3. Stable binding of GFP-tagged WRN.

Supplementary Figure S4. Binomial distributions of GFP photobleaching steps.

Supplementary Figure S5. RPA effect of oligomerization of WRN on forked DNA.

Supplementary Figure S6. Effects of salt and WRN concentrations on the oligomeric states of WRN.

Supplementary Figure S7. Interpretation of fluorescence intensity time traces.

Supplementary Figure S8. GFP counting experiment in the presence of ATPγS.

Supplementary Table S1. Oligonucleotides sequences.

Supplementary Note S1. Single-molecule subunit counting based on GFP photobleaching steps.

Supplementary Figure S1. Purification of WRN.

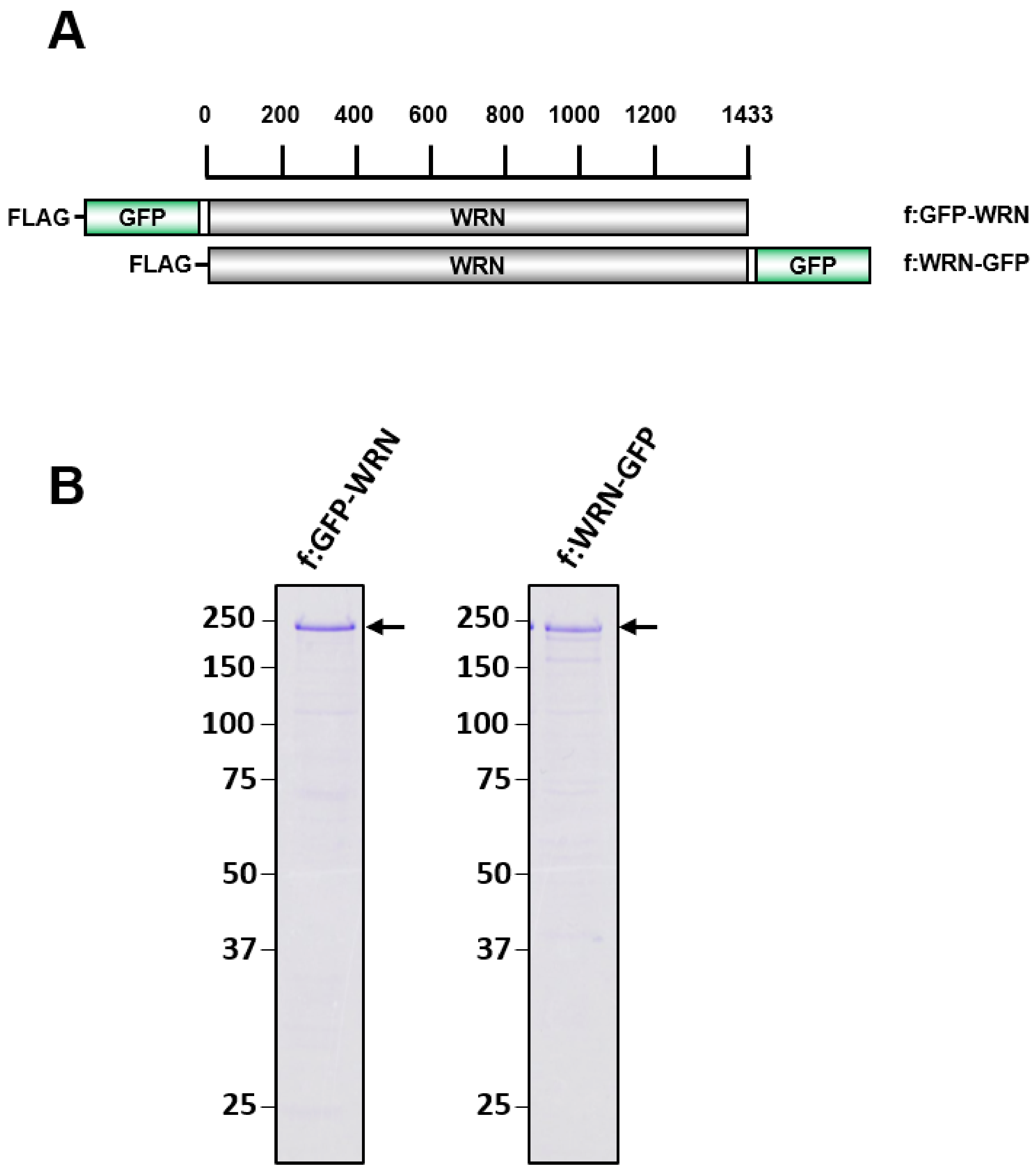

(A) A schematic diagram of N-terminal FLAG-tagged WRN proteins with GFP (B) SDS-PAGE and Coomassie Blue staining of purified FLAG-tagged f:GFP-WRN and f:WRN-GFP proteins. Arrows indicate intact proteins.

Supplementary Figure S2. Comparison of the unwinding and fork regression activities of wild-type WRN and GFP-WRN.

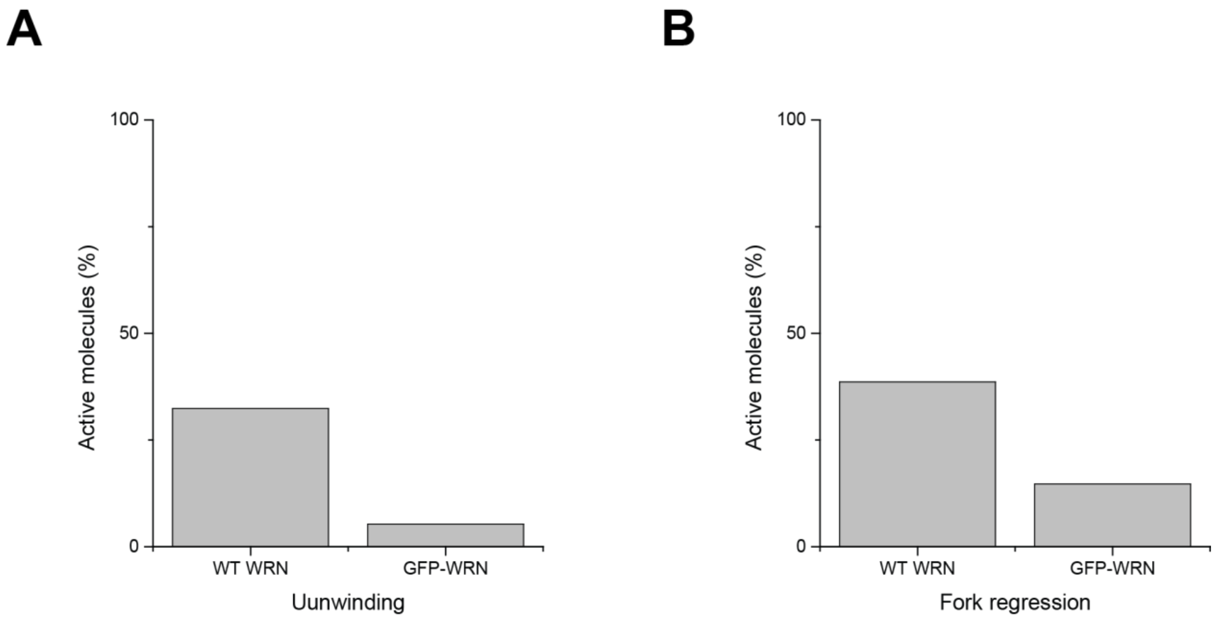

(A) Comparison of unwinding activities of wild-type WRN and GFP-WRN. The single-molecule FRET assay reported in the previous study[1] was used to observe the unwinding events of surface-immobilized forked DNA in the presence of 24 nM WRN and 1 nM RPA. In case of wild-type WRN, 32.4 % (82/253) of DNA substrates exhibited the unwinding behavior whereas 5.3 % (28/532) of molecules were active in case of GFP-WRN. (B) Comparison of fork regression activities of wild-type WRN and GFP-WRN. The single-molecule FRET assay reported in the previous study[2] was used to observe the fork regression events of surface-immobilized model replication forks in the presence of 24 nM WRN. In case of wild-type WRN, 38.6 % (148/383) of replication forks exhibited the fork regression behavior whereas 14.7 % (25/170) of molecules were active in case of GFP-WRN. The lower activities of GFP-WRN than the wild-type WRN are probably due to the steric hindrance of the large GFP domain.

Supplementary Figure S3. Stable binding of GFP-tagged WRN.

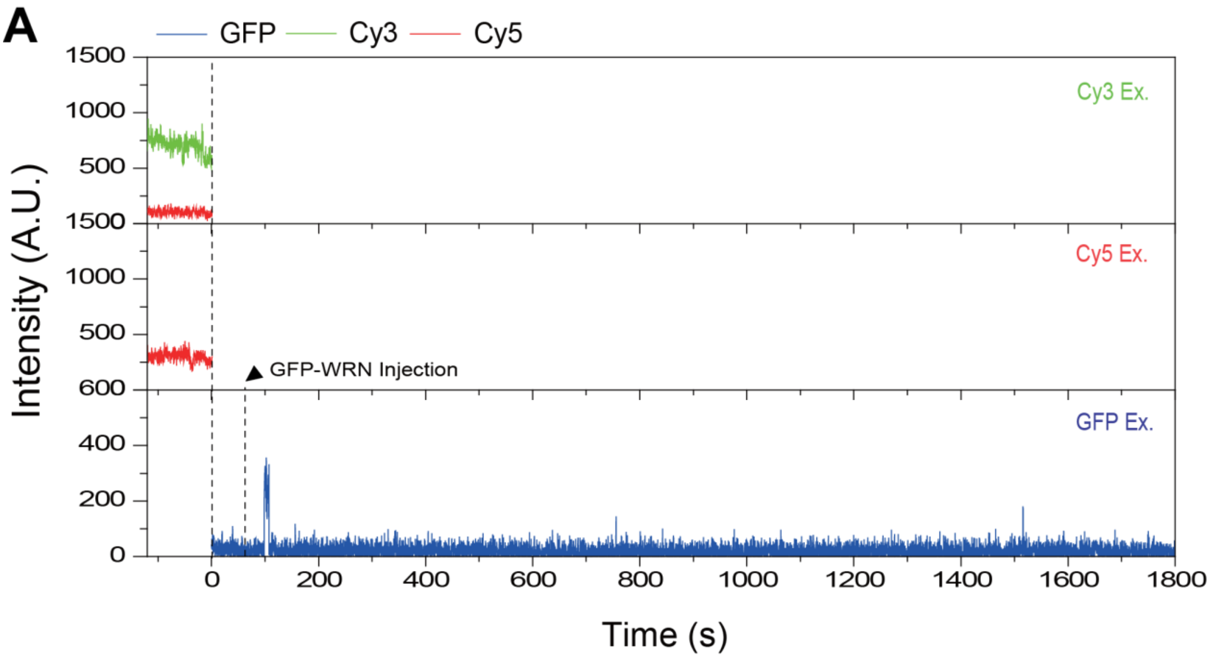

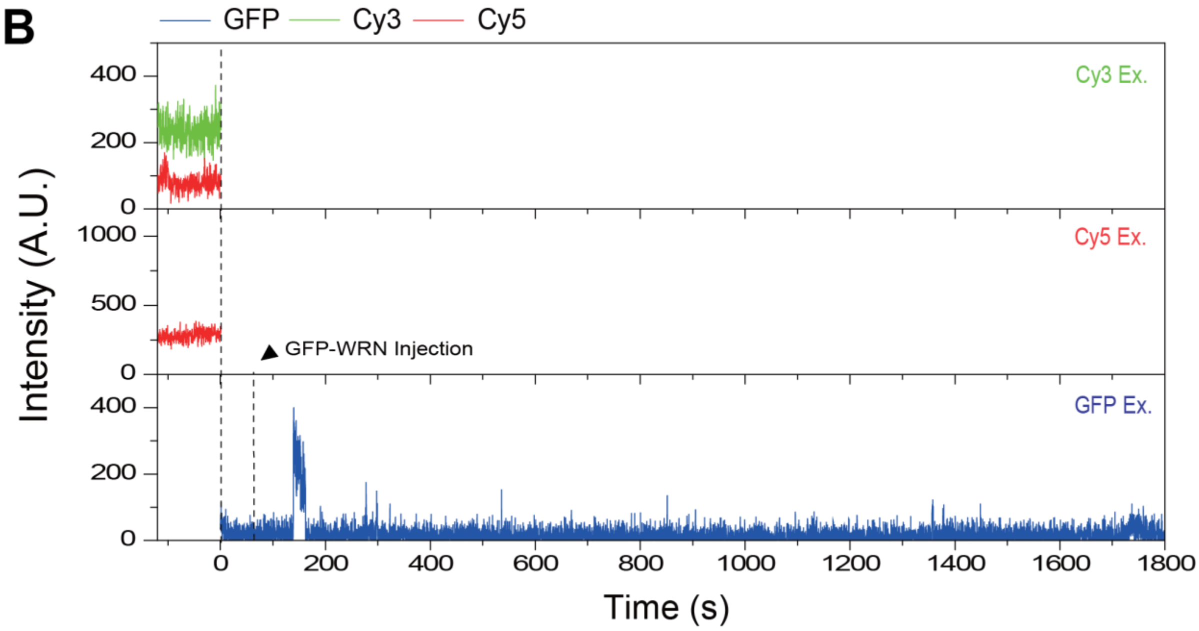

(A) Representative intensity time traces of GFP tagged with WRN (blue) on a forked DNA. (B) Representative intensity time traces of GFP tagged with WRN (blue) on a model replication fork DNA. Cy3 (green) and Cy5 (red) are imaged for co-localization. Black arrow indicates GFP-tagged WRN injection.

Supplementary Figure S4. Binomial distributions of GFP photobleaching steps.

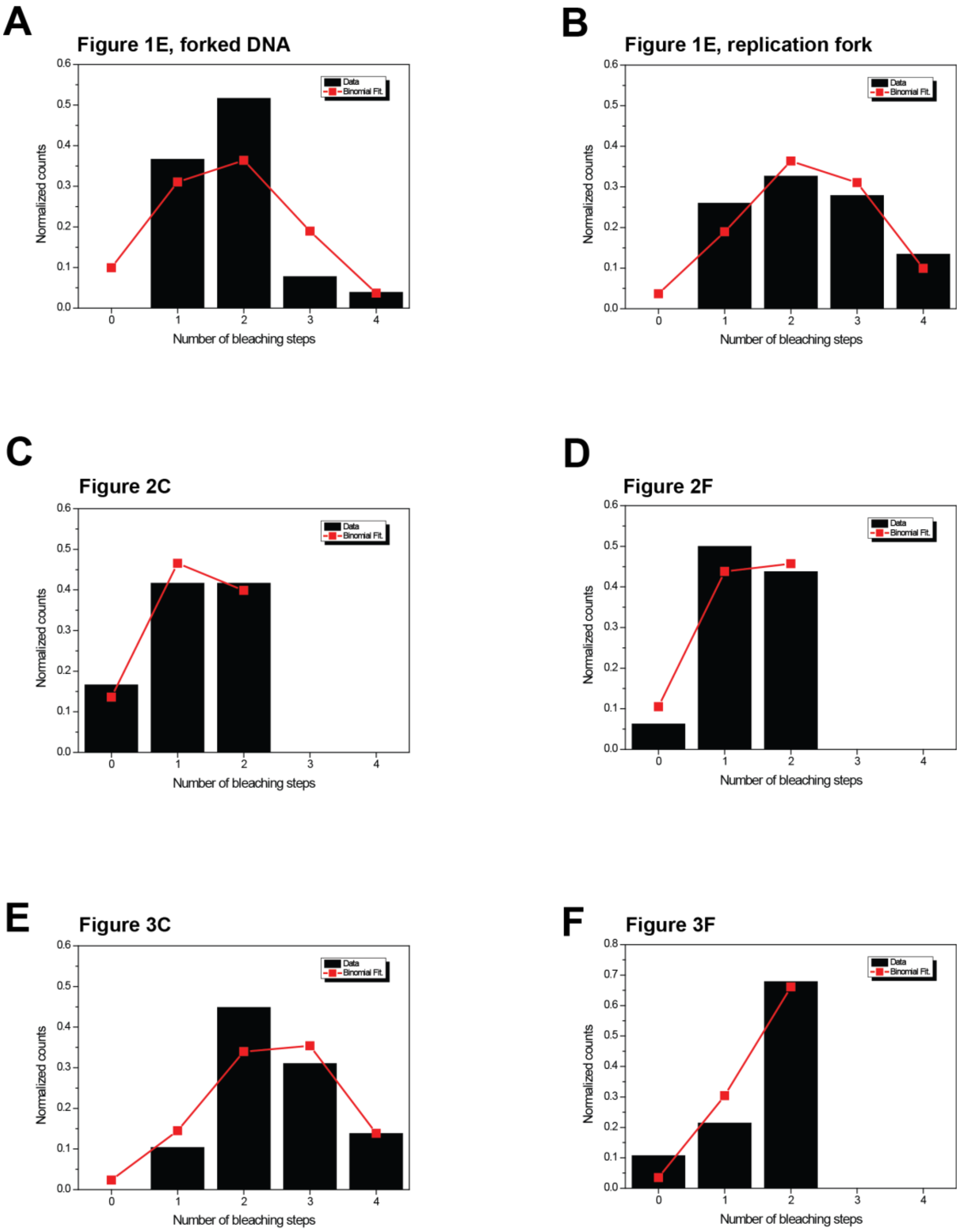

(A) n=4, p=0.44, χ^2=0.240. (B) n=4, p=0.56, χ^2=0.083 (C) n=2, p=0.63, χ^2=0.013. (D) n=2, p=0.67, χ^2=0.027. (E) n=4, p=0.61, χ^2=0.075. (F) n=2, p=0.81, χ^2=0.176. In binomial fitting, n is fixed to the maximum bleaching number.

Supplementary Figure S5. RPA effect of oligomerization of WRN on forked DNA.

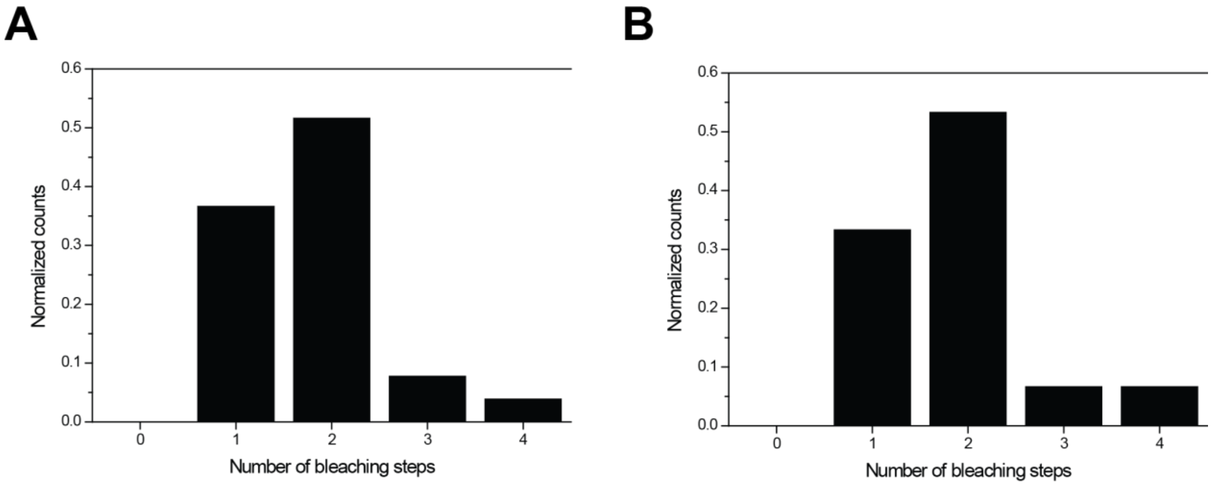

(A) Distribution of bleaching steps of GFP-WRN on forked DNA in the absence of RPA. (B) Distribution of beaching steps of GFP-WRN on forked DNA in the presence of 1 nM RPA.

Supplementary Figure S6. Effects of salt and WRN concentrations on the oligomeric states of WRN.

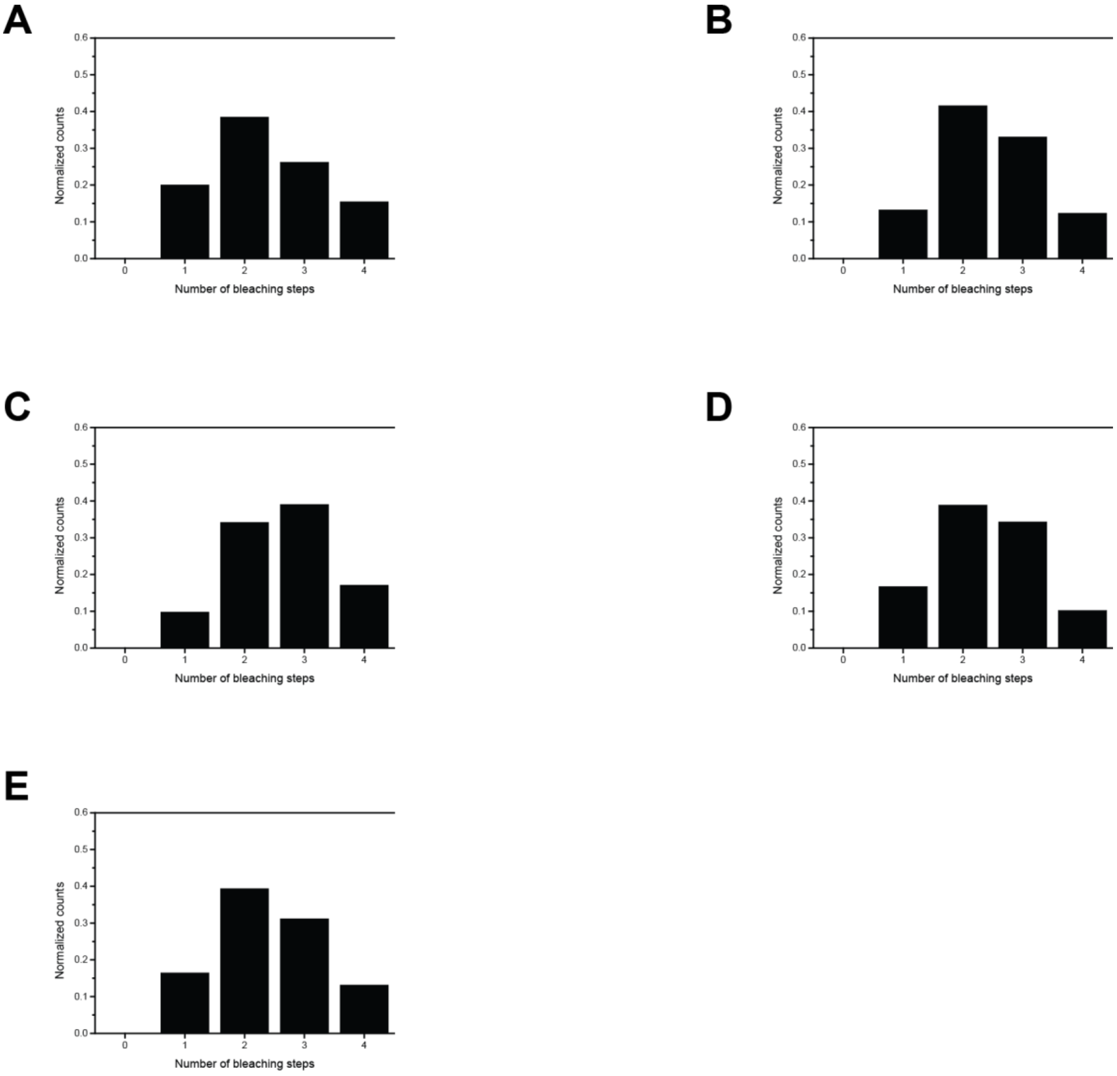

Photobleaching steps of GFP-WRN on the model replication fork were counted in the conditions of (A) 24 nM GFP-WRN and 50 mM NaCl, (B) 48 nM GFP-WRN and 50 mM NaCl, (C) 12 nM GFP-WRN and 50 mM NaCl, (D) 24 nM GFP-WRN and 15 mM NaCl, and (E) 24 nM GFP-WRN and 150 mM NaCl.

Supplementary Figure S7. Interpretation of fluorescence intensity time traces.

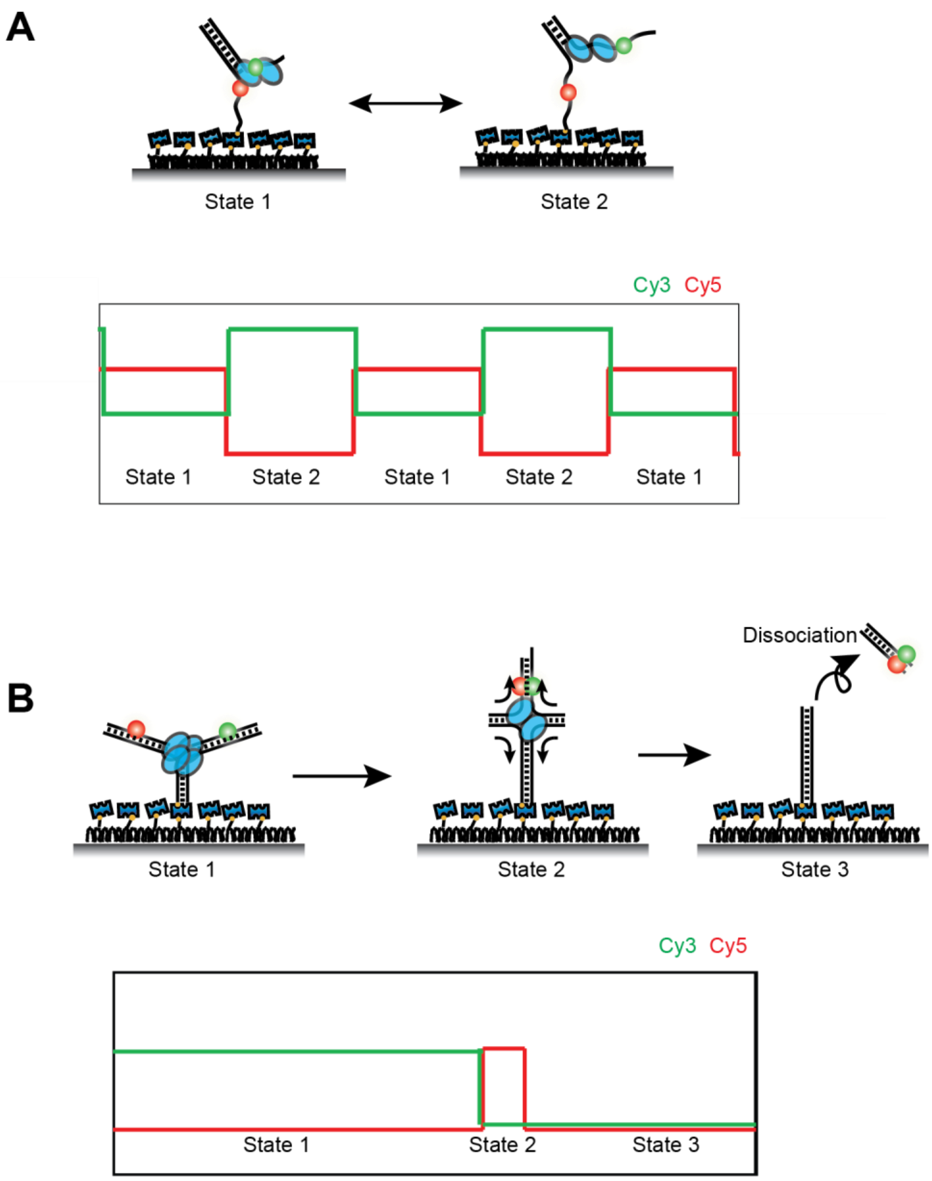

(A) Unwinding experiment. In state 1 (the rewound state) FRET efficiency is high (i.e. Cy5 signal is greater than Cy3) whereas in state 2 (the unwound state) FRET efficiency is low (i.e. Cy3 signal is greater than Cy5). The forked DNA transits between State 1 and State 2 during repetitive unwinding. (B) Fork regression experiment. Before fork regression, FRET efficiency is low whereas FRET efficiency is high when a four-way junction is formed due to fork regression. When fork regression is completed, fluorescence signals of Cy3 and Cy5 disappears due to dissociation of daughter strands.

Supplementary Figure S8. GFP counting experiment in the presence of ATPγS.

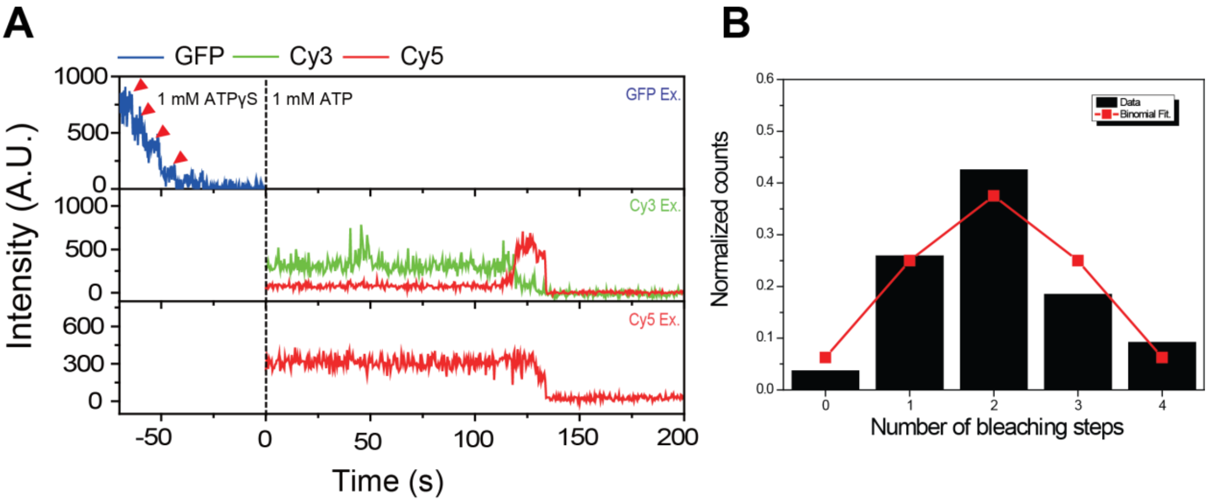

(A) Representative intensity time traces of GFP (blue), Cy3 (green), and Cy5 (red). Red arrow indicates photo-bleaching of single GFP. Dashed line indicates the change from Tirs buffer containing 1mM ATPγS to imaging buffer containing 1mM ATP. (B) Distribution of bleaching steps and its binomial fitting $X \sim B\ (n,\ p)$. n=4, p=0.5, χ^2=0.049. The substrate described in Figure 1B was used for the experiment.

Supplementary Table S1. Oligonucleotides sequences.
RNA sequences are written in lower case. T* or u* means amino C6 dT or amino C6 rU for dye labeling.
All sequences are written 5' to 3' direction.

1. Partial duplex DNA (Figure 1A)

3' overhang _top	80 nt	GAG AAG CAC GCT GCC GAA TTC TAC CAG TGC CTT GCT AGG ACA TGT TTG CCT* TTT TTT TTT TTT TTT TTT TTT TTT TTT TT
5' overhang _bottom	74 nt	Biotin-TTT TTT TTT TTT TTT T T*T TTT TTT GGC AAA CAT GTC CTA GCA AGG CAC TGG TAG AAT TCG GCA GCG TGC TTC TC

2. Replication fork (Figure 1B)

Leading (Daughter)	52 nt	TCG ACA GGT CAT GGC CGT ACA T*GA TAT CCT CGA GCG GTC CTG TTG CAA CTT A
Lagging (Daughter)	60 nt	TCA GAG TGT TAA GTT GCA ACA GGA CCG CTC GAG GAT* ATC ATG TAC GGC CAT GAC CTG TCG
Leading (Parent)	91 nt	Biotin-TGT TAA CCC TAA CCC TAA GAA TTC GGC TTA AGT GAG TGT TAA GTT GCA ACA GGA CCG CTC GAG GAT ATC ATG TAC GGC CAT GAC CTG TCG A
Lagging (Parent)	90 nt	CGA CAG GTC ATG GCC GTA CAT GAT ATC CTC GAG CGG TCC TGT TGC AAC TTA ACA CTC TGA ATA GCC GAA TTC TTA GGG TTA GGG TTA ACA

3. Replication fork with 15 bp non-homologous region (Figure 3D)

Leading (Daughter)	46 nt	TCG ACA GGT CAT GGC CGT* ACA TGA TAT CCT CGA GCG GTC CTG TTG C
Lagging (Daughter)	55 nt	TCA GAG TGT GCA ACA GGA CCG CTC GAG GAT ATC ATG T*AC GAT AGC TTG GCC GCA T
Leading (Parent)	85 nt	Biotin-TGT TAA CCC TAA CCC TAA GAA TTC GGC TTA AGT GAG TGT GCA ACA GGA CCG CTC GAG GAT ATC ATG TAC GGC CAT GAC CTG TCG A
Lagging (Parent)	85 nt	ATG CGG CCA AGC TAT CGT ACA TGA TAT CCT CGA GCG GTC CTG TTG CAC ACT CTG AAT AGC CGA ATT CTT AGG GTT AGG GTT AAC A

4. Replication fork with parent lagging RNA (whole) (Figure 5A)

Leading (Daughter)	52 nt	TCG ACA GGT CAT GGC CGT ACA T*GA TAT CCT CGA GCG GTC CTG TTG CAA CTT A
Lagging (Daughter)	60 nt	TCA GAG TGT TAA GTT GCA ACA GGA CCG CTC GAG GAT* ATC ATG TAC GGC CAT GAC CTG TCG
Leading (Parent)	91 nt	Biotin-TGT TAA CCC TAA CCC TAA GAA TTC GGC TTA AGT GAG TGT TAA GTT GCA ACA GGA CCG CTC GAG GAT ATC ATG TAC GGC CAT GAC CTG TCG A
Lagging (Parent)	90 nt	cga cag guc aug gcc gua cau gau auc cuc gag cgg ucc ugu ugc aac uua aca cuc uga ATA GCC GAA TTC TTA GGG TTA GGG TTA ACA

5. Replication fork with parent lagging RNA (partial) (Figure 5B)

Leading (Daughter)	52 nt	TCG ACA GGT CAT GGC CGT ACA T*GA TAT CCT CGA GCG GTC CTG TTG CAA CTT A
Lagging (Daughter)	60 nt	TCA GAG TGT TAA GTT GCA ACA GGA CCG CTC GAG GAT* ATC ATG TAC GGC CAT GAC CTG TCG
Leading (Parent)	91 nt	Biotin-TGT TAA CCC TAA CCC TAA GAA TTC GGC TTA AGT GAG TGT TAA GTT GCA ACA GGA CCG CTC GAG GAT ATC ATG TAC GGC CAT GAC CTG TCG A
Lagging (Parent)	91 nt	ucg aca ggu cau ggc cgu aca uga uau ccu cga gcg guc cug uug CAA CTT AAC ACT CTG AAT AGC CGA ATT CTT AGG GTT AGG GTT AAC A

6. Replication fork with daughter leading RNA (Figure 5C)

Leading (Daughter)	52 nt	ucg aca ggu cau ggc cgu aca u*ga uau ccu cga gcg guc cug uug caa cuu a
Lagging (Daughter)	60 nt	TCA GAG TGT TAA GTT GCA ACA GGA CCG CTC GAG GAT* ATC ATG TAC GGC CAT GAC CTG TCG
Leading (Parent)	91 nt	Biotin-TGT TAA CCC TAA CCC TAA GAA TTC GGC TTA AGT GAG TGT TAA GTT GCA ACA GGA CCG CTC GAG GAT ATC ATG TAC GGC CAT GAC CTG TCG A
Lagging (Parent)	90 nt	CGA CAG GTC ATG GCC GTA CAT GAT ATC CTC GAG CGG TCC TGT TGC AAC TTA ACA CTC TGA ATA GCC GAA TTC TTA GGG TTA GGG TTA ACA

7. Replication fork with parent lagging RNA (partial) & daughter leading RNA (Figure 5D)

Leading (Daughter)	52 nt	ucg aca ggu cau ggc cgu aca u*ga uau ccu cga gcg guc cug uug caa cuu a
Lagging (Daughter)	60 nt	TCA GAG TGT TAA GTT GCA ACA GGA CCG CTC GAG GAT* ATC ATG TAC GGC CAT GAC CTG TCG
Leading (Parent)	91 nt	Biotin-TGT TAA CCC TAA CCC TAA GAA TTC GGC TTA AGT GAG TGT TAA GTT GCA ACA GGA CCG CTC GAG GAT ATC ATG TAC GGC CAT GAC CTG TCG A
Lagging (Parent)	91 nt	ucg aca ggu cau ggc cgu aca uga uau ccu cga gcg guc cug uug CAA CTT AAC ACT CTG AAT AGC CGA ATT CTT AGG GTT AGG GTT AAC A

8. Replication fork with 15 bp non-homologous region and with daughter lagging RNA (Figure 5E)

Leading (Daughter)	46 nt	TCG ACA GGT CAT GGC CGT* ACA TGA TAT CCT CGA GCG GTC CTG TTG C
Lagging (Daughter)	55 nt	uca gag ugu gca aca gga ccg cuc gag gau auc aug u*ac gau agc uug gcc gca u
Leading (Parent)	85 nt	Biotin-TGT TAA CCC TAA CCC TAA GAA TTC GGC TTA AGT GAG TGT GCA ACA GGA CCG CTC GAG GAT ATC ATG TAC GGC CAT GAC CTG TCG A
Lagging (Parent)	85 nt	ATG CGG CCA AGC TAT CGT ACA TGA TAT CCT CGA GCG GTC CTG TTG CAC ACT CTG AAT AGC CGA ATT CTT AGG GTT AGG GTT AAC A

9. Replication fork with 15 bp non-homologous region and with parent leading RNA (Figure 5F)

Leading (Daughter)	46 nt	TCG ACA GGT CAT GGC CGT* ACA TGA TAT CCT CGA GCG GTC CTG TTG C
Lagging (Daughter)	55 nt	TCA GAG TGT GCA ACA GGA CCG CTC GAG GAT ATC ATG T*AC GAT AGC TTG GCC GCA T
Leading (Parent)	85 nt	Biotin-TGT TAA CCC TAA CCC TAA GAA TTC GGC TTA AGT GAG TGT gca aca gga ccg cuc gag gau auc aug uac ggc cau gac cug ucg a
Lagging (Parent)	85 nt	ATG CGG CCA AGC TAT CGT ACA TGA TAT CCT CGA GCG GTC CTG TTG CAC ACT CTG AAT AGC CGA ATT CTT AGG GTT AGG GTT AAC A

10. Replication fork with 15 bp non-homologous region with daughter lagging RNA & parent leading RNA (Figure 5G)

Leading (Daughter)	46 nt	TCG ACA GGT CAT GGC CGT* ACA TGA TAT CCT CGA GCG GTC CTG TTG C
Lagging (Daughter)	55 nt	uca gag ugu gca aca gga ccg cuc gag gau auc aug u*ac gau agc uug gcc gca u
Leading (Parent)	85 nt	Biotin-TGT TAA CCC TAA CCC TAA GAA TTC GGC TTA AGT GAG TGT gca aca gga ccg cuc gag gau auc aug uac ggc cau gac cug ucg a
Lagging (Parent)	85 nt	ATG CGG CCA AGC TAT CGT ACA TGA TAT CCT CGA GCG GTC CTG TTG CAC ACT CTG AAT AGC CGA ATT CTT AGG GTT AGG GTT AAC A

Supplementary Note S1. Single-molecule subunit counting based on GFP photobleaching steps.

Single molecule photobleaching is a powerful tool for determining the stoichiometry and oligomerization of protein complexes[3-7]. By attaching fluorophores to proteins, the number of associated subunits in a complex can be deduced by counting photobleaching steps. Because some bleaching steps are unobserved (mainly due to the nonfluorescent state of fluorophores[3, 8]), the step number distribution should be binomial in case of homogeneous population. A number of studies used this technique to examine the stoichiometry of proteins such as ligand-gated ion channels[6], voltage-gated ion channels[9], and helicase loader protein[10]. The method comprises attaching a fluorescent probe (typically GFP or its variants) to protein subunit and imaging single molecules. After the excitation fluorophores by laser, the fluorophores will sequentially bleach, resulting in a step-wise decrease of the intensity of fluorescence. Then, by simply counting the number of the bleaching steps, we can observe how many subunits, n, were associated in the observed complex. However, because there is a probability, 1-p, that any given fluorophore is inactive due to bleaching in the preparation steps, misfolding, or any other reason, we observe the number of photobleaching steps of each protein less than or equal to the actual subunit number, n. The parameter p is the probability of successfully observing each possible photobleaching event. Therefore, the resulting observations of a homogeneous population are binomially distributed ($B(n,p)$) where the maximum number of observed photobleaching steps is assigned to n[3].

References

1. Lee, M. et al. Multiple RPAs make WRN syndrome protein a superhelicase. *Nucleic acids research* **46**, 4689-4698 (2018).
2. Shin, S. et al. Active control of repetitive structural transitions between replication forks and Holliday junctions by Werner syndrome helicase. *Structure* **24**, 1292-1300 (2016).
3. Ulbrich, M.H. & Isacoff, E.Y. Subunit counting in membrane-bound proteins. *Nature methods* **4**, 319-321 (2007).
4. Ulbrich, M.H. & Isacoff, E.Y. Rules of engagement for NMDA receptor subunits. *Proceedings of the National Academy of Sciences* **105**, 14163-14168 (2008).
5. Reiner, A., Arant, R.J. & Isacoff, E.Y. Assembly stoichiometry of the GluK2/GluK5 kainate receptor complex. *Cell reports* **1**, 234-240 (2012).
6. Yu, Y. et al. Molecular mechanism of the assembly of an acid-sensing receptor ion channel complex. *Nature communications* **3**, 1-11 (2012).
7. Coste, B. et al. Piezo proteins are pore-forming subunits of mechanically activated channels. *Nature* **483**, 176-181 (2012).
8. Garcia-Parajo, M., Koopman, M., Van Dijk, E., Subramaniam, V. & Van Hulst, N. The nature of fluorescence emission in the red fluorescent protein DsRed, revealed by single-molecule detection. *Proceedings of the National Academy of Sciences* **98**, 14392-14397 (2001).
9. Nakajo, K., Ulbrich, M.H., Kubo, Y. & Isacoff, E.Y. Stoichiometry of the KCNQ1-KCNE1 ion channel complex. *Proceedings of the National Academy of Sciences* **107**, 18862-18867 (2010).
10. Arumugam, S.R., Lee, T.-H. & Benkovic, S.J. Investigation of stoichiometry of T4 bacteriophage helicase loader protein (gp59). *Journal of Biological Chemistry* **284**, 29283-29289 (2009).
